# ISIT-QA: In Silico Imaging Trial to Evaluate a Low-Count Quantitative SPECT Method Across Multiple Scanner–Collimator Configurations for ^223^Ra-Based Radiopharmaceutical Therapies

**DOI:** 10.2967/jnumed.123.266719

**Published:** 2024-05

**Authors:** Zekun Li, Nadia Benabdallah, Jingqin Luo, Richard L. Wahl, Daniel L.J. Thorek, Abhinav K. Jha

**Affiliations:** 1 Department of Biomedical Engineering, Washington University, St. Louis, Missouri;; 2 Mallinckrodt Institute of Radiology, Washington University, St. Louis, Missouri;; 3 Program in Quantitative Molecular Therapeutics, Washington University, St. Louis, Missouri;; 4 Siteman Cancer Center, Washington University, St. Louis, Missouri;; 5 Division of Public Health Sciences, Department of Surgery, Washington University, St. Louis, Missouri; and; 6 Division of Biostatistics, Washington University, St. Louis, Missouri

**Keywords:** in silico imaging trial, low-count quantitative SPECT, theranostics, reproducibility, α-particle–emitting radiopharmaceutical therapy

## Abstract

Personalized dose-based treatment planning requires accurate and reproducible noninvasive measurements to ensure safety and effectiveness. Dose estimation using SPECT is possible but challenging for alpha (α)-particle–emitting radiopharmaceutical therapy (α-RPT) because of complex γ-emission spectra, extremely low counts, and various image-degrading artifacts across a plethora of scanner–collimator configurations. Through the incorporation of physics-based considerations and skipping of the potentially lossy voxel-based reconstruction step, a recently developed projection-domain low-count quantitative SPECT (LC-QSPECT) method has the potential to provide reproducible, accurate, and precise activity concentration and dose measures across multiple scanners, as is typically the case in multicenter settings. To assess this potential, we conducted an in silico imaging trial to evaluate the LC-QSPECT method for a ^223^Ra-based α-RPT, with the trial recapitulating patient and imaging system variabilities. **Methods:** A virtual imaging trial titled In Silico Imaging Trial for Quantitation Accuracy (ISIT-QA) was designed with the objectives of evaluating the performance of the LC-QSPECT method across multiple scanner–collimator configurations and comparing performance with a conventional reconstruction–based quantification method. In this trial, we simulated 280 realistic virtual patients with bone-metastatic castration-resistant prostate cancer treated with ^223^Ra-based α-RPT. The trial was conducted with 9 simulated SPECT scanner–collimator configurations. The primary objective of this trial was to evaluate the reproducibility of dose estimates across multiple scanner–collimator configurations using LC-QSPECT by calculating the intraclass correlation coefficient. Additionally, we compared the reproducibility and evaluated the accuracy of both considered quantification methods across multiple scanner–collimator configurations. Finally, the repeatability of the methods was evaluated in a test–retest study. **Results:** In this trial, data from 268 ^223^RaCl_2_ treated virtual prostate cancer patients, with a total of 2,903 lesions, were used to evaluate LC-QSPECT. LC-QSPECT provided dose estimates with good reproducibility across the 9 scanner–collimator configurations (intraclass correlation coefficient > 0.75) and high accuracy (ensemble average values of recovery coefficients ranged from 1.00 to 1.02). Compared with conventional reconstruction-based quantification, LC-QSPECT yielded significantly improved reproducibility across scanner–collimator configurations, accuracy, and test–retest repeatability (P<0.01). **Conclusion:** LC-QSPECT provides reproducible, accurate, and repeatable dose estimations in ^223^Ra-based α-RPT as evaluated in ISIT-QA. These findings provide a strong impetus for multicenter clinical evaluations of LC-QSPECT in dose quantification for α-RPTs.

## Visual Abstract

**Figure:**
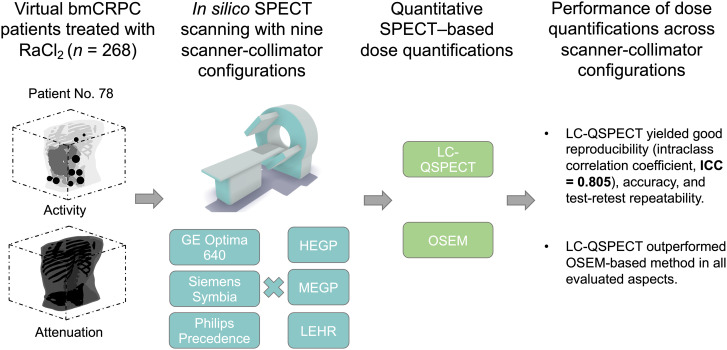

Targeted α-therapy is gaining increasing clinical significance (1–3). Because of their short emission range and high linear energy transfer, α-particles can effectively ablate the regions where they are deposited, with minimal damage to adjacent tissues (3). However, the systemically administered radiopharmaceuticals distribute throughout the patient, accumulating to unknown levels at sites of disease and in radiosensitive vital organs. Thus, it is important to quantify the absorbed doses of the lesions and different organs of the patient treated with these potent agents. Absorbed dose measurement to diseased and healthy tissues is the standard of care for conventional external beam radiotherapy. Such dose quantification in radiopharmaceutical therapies can be beneficial by allowing for adaption of treatment regimens, prediction of therapy outcomes, and monitoring of adverse events (4).

Therapeutic α-particle-emitting isotopes often also emit γ-ray photons detectable by γ-cameras. This provides a mechanism to quantify the absorbed dose by measuring the activity uptake in organs and lesions using quantitative SPECT methods. A major challenge in α-particle–emitting radiopharmaceutical therapy (α-RPT) SPECT is the extremely low number of detected photon counts. This number can be several orders of magnitude lower than in conventional diagnostic SPECT (5). Consequently, conventional quantitative SPECT (QSPECT) methods yield low precision and accuracy in estimating uptake, even with fine-tuned protocols (6–9). Another challenge lies in modeling and compensating for the complicated SPECT physics. α-emitting isotopes, such as ^223^Ra, ^225^Ac, and ^227^Th, have a complex decay chain with multiple progenies (5*,*^10^). The γ-spectra of those isotopes usually consist of multiple photopeaks, which further complicates modeling and compensation of SPECT physics including attenuation, scatter, collimator–detector response, and system resolution (11). Inaccurate modeling of the SPECT physics results in systematic variability of uptake quantification across different SPECT scanner–collimator configurations, including from different vendors, hardware specifications, and collimators (12). Previous investigations indicate that even for conventional (high-count) SPECT, differences in uptake estimates between scanner–collimator configurations may reach up to 41% of the actual value (12–14). However, such systematic variability of quantifications remains unexplored in the context of α-RPTs.

Unreliable activity quantification results in a lack of reliability in dose quantification. This significantly impedes the advancement of personalized α-RPT regimens (15) and hinders the computing of robust dose–response relationships for treatment optimization and drug development. Further, variability across SPECT scanner–collimator configurations complicates the combining and comparing of data across instruments and across centers (12–14*,*^16^). Critically these inconsistencies hinder multiinstitutional data comparison, a hallmark of the scientific approach to medical advancement. Finally, errors in quantifying absorbed doses in lesions and at-risk organs are undesirable given the high potential tissue toxicity of α-particles (17). This can also lead to an erroneous diagnosis and therapeutic outcome evaluation using radiobiologic models (18).

A recently developed low-count QSPECT (LC-QSPECT) method has demonstrated the ability to yield precise and accurate estimates of regional uptake in α-RPTs. The method estimates the uptake directly from projection data, avoiding information losses inherent in tomographic voxel-based reconstruction processes. In evaluations based on a single scanner–collimator configuration, the method yielded nearly unbiased absolute quantification of regional uptake, with estimate variance being close to the theoretically lowest possible values, outperforming conventional QSPECT methods (9). Notably, this LC-QSPECT method compensates for the physics of given SPECT scanner–collimator configurations directly and accurately in the quantification process. Therefore, we hypothesized that LC-QSPECT could deliver reproducible, repeatable, and accurate dose measurements across multiple SPECT scanner–collimator configurations, making it a compelling candidate for further multiple-center clinical evaluations and potential applications.

Clinical evaluation of the reproducibility across scanner–collimator configurations, accuracy, and repeatability of dose measurements with QSPECT methods requires that a patient administered α-RPT be imaged across multiple SPECT/CT scanner–collimator configurations or multiple times with the same configuration. This process in practice would lead to a substantial CT radiation dose, has ethical issues, is logistically challenging, is time-consuming, and, even if possible, would suffer from the limitation of lack of ground truth. The emerging virtual imaging trial paradigm provides a mechanism to overcome these limitations, enabling rigorous, objective evaluation of imaging technology performance in simulated clinical scenarios that account for patient population variability and system physics (19–21). For instance, the seminal VICTRE in silico trial demonstrated the utility of virtual imaging trials for regulatory evidence of imaging technologies (20). In this study, we conducted a virtual imaging trial (In Silico Imaging Trial for Quantitation Accuracy [ISIT-QA]) that recapitulates patient and imaging system variabilities to evaluate LC-QSPECT across multiple scanner–collimator configurations on the task of dose estimation in α-RPTs. Additionally, the method was compared to a conventional ordered subset expectation maximization (OSEM) method that reconstructed the images over a voxelized grid. We refer to this method as the OSEM method in the rest of the article.

## MATERIALS AND METHODS

### Study Design

**FIGURE 1.**
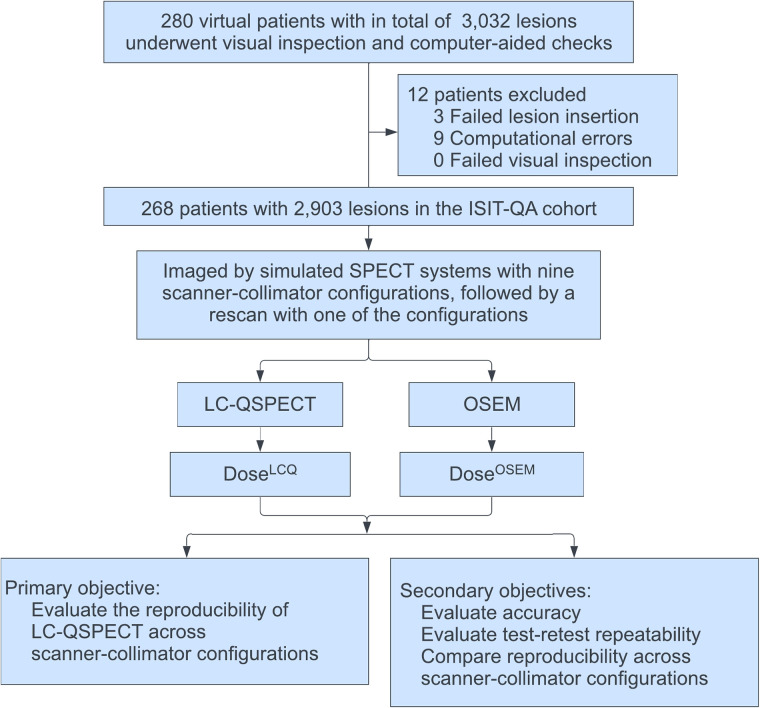
Overall design of ISIT-QA.

ISIT-QA aimed to assess the performance of the SPECT methods for estimating tissue- and lesion-absorbed dose from ^223^RaCl_2_, the first α-RPT approved by the U.S. Food and Drug Administration for the treatment of bone-metastatic castration-resistant prostate cancer (1). Figure 1 shows the overall trial design. This institutional review board–exempt study (study identification no. 202303134) was conducted at Washington University in St. Louis.

In this trial, we simulated 280 realistic and diverse bone-metastatic castration-resistant prostate cancer patients intravenously administered standard ^223^Ra activities. Virtual patients were excluded from the imaging cohort if errors arose during phantom generations. As in the VICTRE trial, exclusion criteria were enforced by software error messages, computer-aided checks, and visual inspections (20). Eligible virtual patients were imaged with 9 scanner–collimator configurations in silico, followed by a rescan using one of the scanner–collimator configurations. We then applied LC-QSPECT and the OSEM method to estimate the uptake and then the absorbed doses of different volumes of interest (VOIs) of the patients.

The primary objective of this trial was to evaluate the reproducibility of estimated doses obtained with LC-QSPECT across multiple scanner–collimator configurations. The secondary objectives included comparing the reproducibility of LC-QSPECT across scanner–collimator configurations with the OSEM method, evaluating the accuracy of the QSPECT methods across multiple scanner–collimator configurations, and assessing the test–retest repeatability of the methods.

### Trial Population

**FIGURE 2.**
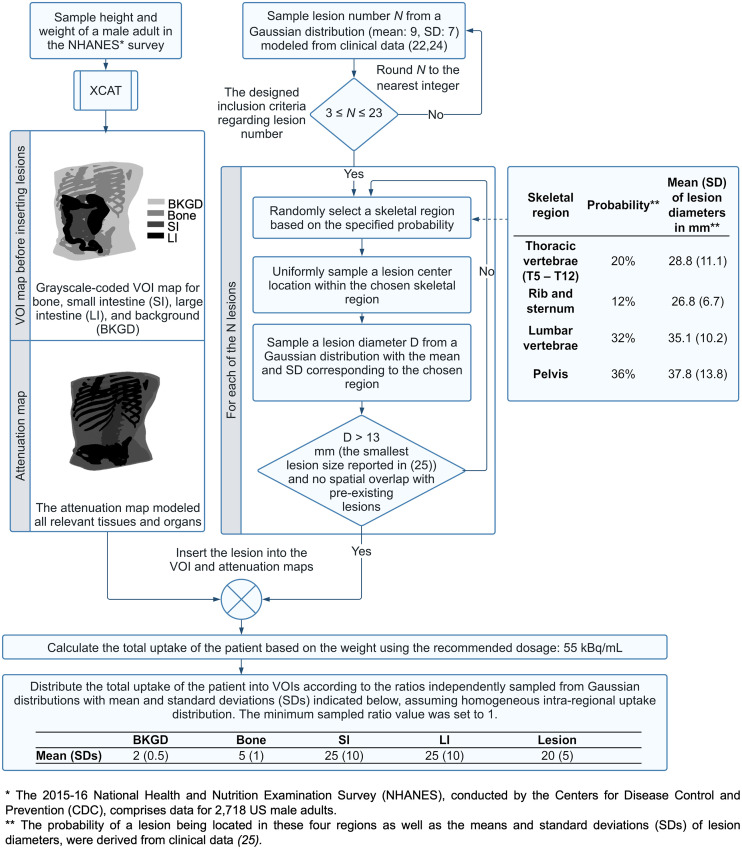
Virtual patient generation process.

The process to generate the trial population is shown in Figure 2 and briefly summarized here, with details provided in Supplemental Section 1 (supplemental materials are available at http://jnm.snmjournals.org) (7*,*^9^*,*^22^–^30^). To simulate virtual patients with bone-metastatic castration-resistant prostate cancer, we first generated 280 healthy virtual U.S. male phantoms using the Extended Cardiac-Torso (XCAT) (23*,*^28^). The heights and weights of the virtual patients were directly sampled from data on 2,718 U.S. men published by the Centers for Disease Control and Prevention. Then, we inserted lesions into each phantom, simulating realistic disease characteristics including lesion count, lesion diameters, and the spatial distribution of the lesions, all of which were derived from multiple clinical studies (22*,*^24^–^27^). A rigorous process was followed to insert the lesions, as outlined in the flowchart in Figure 2. Informed by distribution data from preclinical and clinical evaluations of ^223^Ra (26*,*^27^), we considered 5 categories of VOIs in the patients: lesions, bone, small intestine, large intestine, and background. Following a previously published approach (9), we modeled homogeneous activity distribution within each VOI, with mean uptake values determined as illustrated in Figure 2.

### Imaging Protocol

We simulated the clinical scenario in which patients received a single intravenous 55 kBq/kg injection of ^223^Ra, aligning with the recommended dosage (31). On the basis of pharmacokinetic imaging studies demonstrating rapid blood clearance, we assumed patients would exhibit a representative ^223^Ra biodistribution, as indicated by the activity maps of the virtual patients, 30 min after administration (26). Thus, this time point was selected for imaging the patients.

SPECT scans were generated using SIMIND, a well-validated Monte Carlo simulation software (9*,*^32^–^34^). We further validated our SIMIND-based approach for simulating the imaging of ^223^Ra across various scanner–collimator configurations using physical phantom studies (Supplemental Section 7; Supplemental Figs. 1–5; Supplemental Tables 1–2 (9*,*^35^)). The entire patient cohort was imaged using 9 simulated SPECT scanner–collimator configurations: 3 scanners (GE Healthcare Optima 640, Siemens Symbia, and Philips Precedence), each with 3 collimator configurations (high-energy general-purpose, medium-energy general-purpose, and low-energy high-resolution). Details on simulating the imaging processes are provided in Supplemental Section 2 (6*,*^9^*,*^32^–^34^).

After completion of the primary imaging study, patients underwent a second scan 30 min after the end of the first scan, using the simulated Optima 640 scanner with a high-energy general-purpose collimator. We simulated the physical decay of ^223^Ra (half-life, 11.4 d) and a constant biologic distribution of the isotope over the 30-min duration.

We assumed a CT scan was acquired before each SPECT scan, yielding the required VOI definitions for the QSPECT methods. To reduce unrelated confounding factors, these VOI definitions were assumed to be accurate. Supplemental Section 8 (36*,*^37^) explores the impact of potential inaccuracies in the used VOI definitions, caused by rigid or nonrigid patient body transformations, on the performance of the QSPECT methods (Supplemental Figs. 6–7). Additionally, Supplemental Section 9 evaluates the impact of misalignment of VOI definitions in a test–retest study scenario (Supplemental Fig. 8; Supplemental Table 3).

### Activity and Dose Quantification: LC-QSPECT Method and Comparison Studies

We first estimated the uptake of ^223^Ra in lesions and organs of patients using both LC-QSPECT and OSEM methods. The theory and implementation of the LC-QSPECT method were detailed by Li et al. (9) and are presented in Supplemental Section 3 (6*,*^8^*,*^9^*,*^13^*,*^32^–^34^*,*^38^–^40^). Implementation of the OSEM approach (41) is described in Supplemental Section 4 (41–44) and is similar to that presented by Li et al. (9). Briefly, the LC-QSPECT method is a maximum-likelihood technique (38) to estimate the mean uptake in various VOIs directly from SPECT projections, accounting for various image-degrading effects in α-particle SPECT, including the complicated SPECT and isotope physics (9). The OSEM approach adopts the conventional route of first reconstructing the image over a voxelized grid, compensating for all the relevant image-degrading effects in SPECT, followed by averaging the uptake within the VOIs defined over the reconstructed image.

Subsequently, the regional absorbed doses were calculated using output obtained from both methods on the basis of the estimated uptake using the MIRD schema, adopting a single-time-point dosimetry (45) model and assuming no redistribution of ^223^Ra or its progeny. A detailed description of the dose calculation process is in Supplemental Section 5 (45*,*^46^).

### Statistical Considerations

For the primary objective, that is, to evaluate the reproducibility of the LC-QSPECT method, we calculated the intraclass correlation coefficient (ICC) of the estimated doses across the 9 scanner–collimator configurations. The ICC was calculated using the single-measurement, absolute-agreement, 2-way mixed-effects model and interpreted according to the guidelines proposed by Koo and Li (47). We calculated the sample size to achieve sufficient precision in estimating the interscanner–collimator-configuration ICC with a 2-sided 95% CI width of 0.1. We targeted an ICC of at least 0.5, ranging from 0.5 to 0.9. The total patient count was calculated as 267, 230, 168, 94, and 30 for ICCs of 0.5, 0.6, 0.7, 0.8, and 0.9, respectively. To account for an approximately 5% drop-out rate, we simulated 280 virtual patients. In addition, we also examined the interscanner- and intercollimator-configuration reproducibility of the method by assessing the ICC of the estimates across the 3 scanners with a high-energy general-purpose collimator and the ICC of estimates across the 3 collimator configurations with the Optima 640 scanner, respectively.

For our secondary objectives, we quantified and compared the accuracy and test–retest repeatability of both QSPECT methods. We used the recovery coefficient (RC)—defined as the ratio of the estimated value to the ground truth—for measuring accuracy. For quantifying test–retest repeatability, we used the within-subject coefficient of variation (CV). To assess the agreement of the estimated doses in the test–retest study, we conducted a Bland–Altman analysis using the percentage difference (defined as the difference between test and retest estimates normalized by their mean (48)), considering the heteroscedasticity of the estimates in this trial. Additionally, we compared the reproducibility of the LC-QSPECT and OSEM methods across all considered scanner–collimator configurations, across the 3 scanners with a high-energy general-purpose collimator, and across the 3 collimator configurations with an Optima 640 scanner, using the ensemble-averaged CV. Details on computing the figures of merit are provided in Supplemental Section 6. Paired-sample *t* tests were used to determine whether there were statistically significant differences between the performance of LC-QSPECT and OSEM methods.

## RESULTS

### Trial Accrual

**FIGURE 3.**
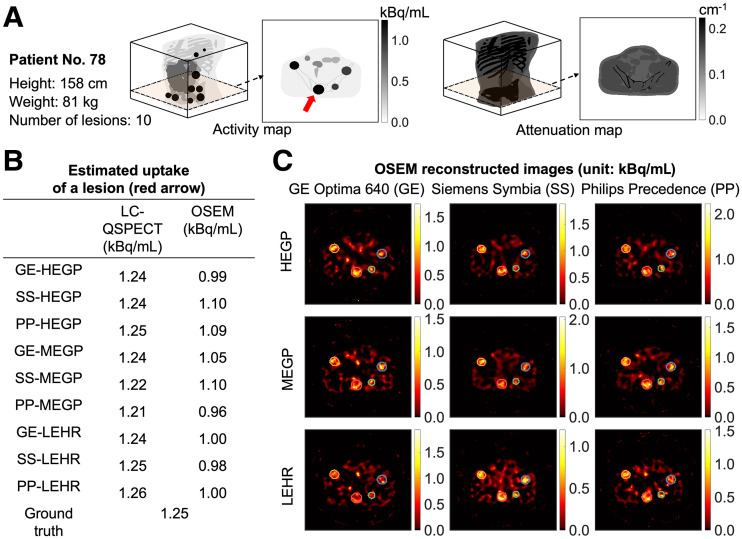
(A) Three-dimensional visualization of activity and attenuation maps of 78th patient in cohort and representative slice from each map. Index lesion is indicated by red arrow. (B) Estimated uptake of index lesion using LC-QSPECT and OSEM methods for this patient. Ground truth uptake also provided. (C) OSEM-reconstructed images of representative slice with each of 9 SPECT scanner–collimator configurations. Lesion boundaries used for OSEM estimations are provided, and they are derived from same lesion masks used in LC-QSPECT method. HEGP = high-energy general-purpose; LEHR = low-energy high-resolution; MEGP = medium-energy general-purpose.

In ISIT-QA, 12 of 280 virtual patients were excluded, leading to a final cohort of 268 virtual patients with a total of 2,903 lesions. The demographics and disease characteristics of these patients are shown in Table 1. Activity and attenuation maps for a representative virtual patient are shown in Figure 3A. Figure 3B presents estimated uptake of an index lesion (indicated in Fig. 3A) using both QSPECT methods. The corresponding OSEM-reconstructed images with different scanner–collimator configurations are shown in Figure 3C. Data generated as part of this virtual imaging trial are available at https://wustl.box.com/v/ISITQA-VIT-data.

**TABLE 1.** Demographics and Disease Characteristics of Patients in Cohort

Characteristic	Data
Number of patients	268
Extent of disease	
<6 metastases	49 (18.3%)
6–20 metastases	212 (79.1%)
>20 metastases	7 (2.6%)
Total number of lesions	2,903
Lesion locations	
Thoracic vertebrae (T5–T12)	569 (19.6%)
Rib and sternum	427 (14.7%)
Lumbar vertebrae	928 (32.0%)
Pelvis	979 (33.7%)
Lesion diameter	
<25 mm	566 (19.5%)
25–35 mm	975 (33.6%)
>35 mm	1,362 (46.9%)
Patient height (cm)	173.1 (7.8)
Patient weight (kg)	86.6 (21.1)
Mean uptake (kBq/mL)	
Background	0.06 (0.015)
Bone	0.16 (0.043)
Small intestine	0.77 (0.26)
Large intestine	0.77 (0.30)
Lesions	0.64 (0.21)

Data are count and percentage for categoric characteristics and mean and SD for quantitative characteristics.

### Reproducibility Across Scanner–Collimator Configurations

Table 2 shows the ICCs evaluating the reproducibility of the LC-QSPECT method across all considered scanner–collimator configurations, across the 3 scanners with a high-energy general-purpose collimator, and across the 3 collimator configurations with an Optima 640 scanner. The CIs of all the ICC values were between 0.76 and 0.82, demonstrating that the LC-QSPECT method has good reproducibility (47).

**TABLE 2.** ICC of Estimated Doses Using LC-QSPECT Method Across All Considered Scanner–Collimator Configurations, Across 3 Scanners with High-Energy General-Purpose Collimator, and Across 3 Collimator Configurations with Optima 640 Scanner

		95% CI	
Dose estimates across…	ICC	Lower limit	Upper limit
Scanner–collimator configurations	0.805	0.797	0.812
Scanners	0.774	0.763	0.784
Collimator configurations	0.789	0.779	0.798

**FIGURE 4.**
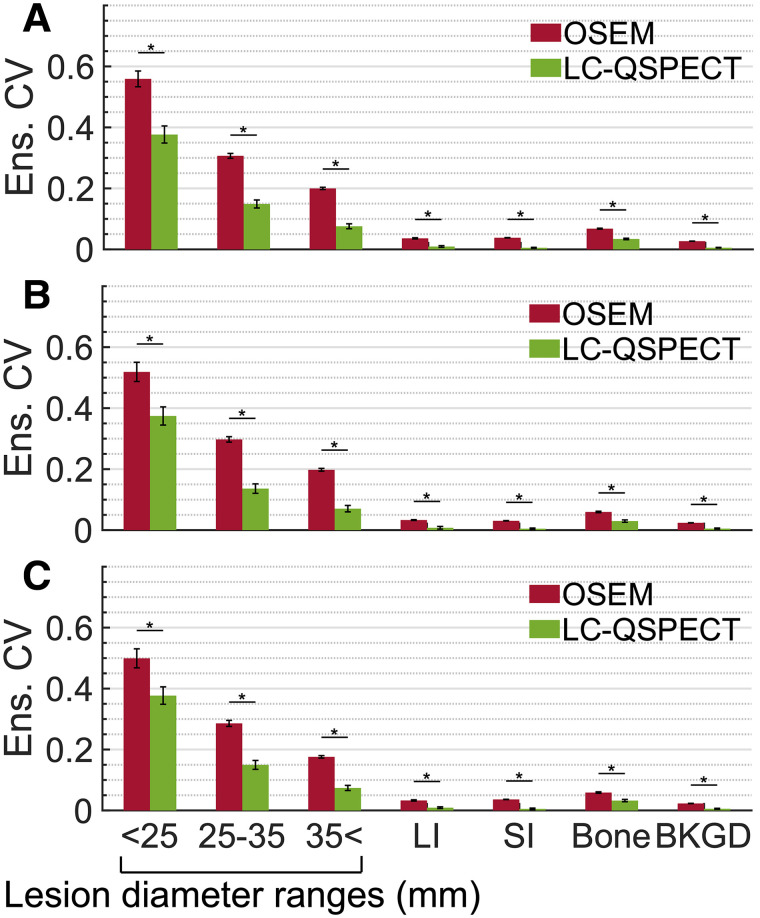
Ensemble-averaged (Ens.) CV for each group of VOIs evaluating reproducibility of estimated doses across all considered scanner–collimator configurations (A), 3 scanners with high-energy general-purpose collimator (B), and 3 collimators with Optima 640 scanner (C). Error bars show standard error (SE) of ensemble-averaged CVs. *P < 0.01. BKGD = background; LI = large intestine; SI = small intestine.

Figures 4A–4C compare the ensemble-averaged CVs of the LC-QSPECT and OSEM methods, evaluating the reproducibility across the scanner–collimator configurations, scanners, and collimator configurations, respectively. Overall, for all groups of VOIs, the LC-QSPECT method yielded ensemble-averaged CVs below 0.4 and consistently outperformed the OSEM method (P<0.01).

### Accuracy

**FIGURE 5.**
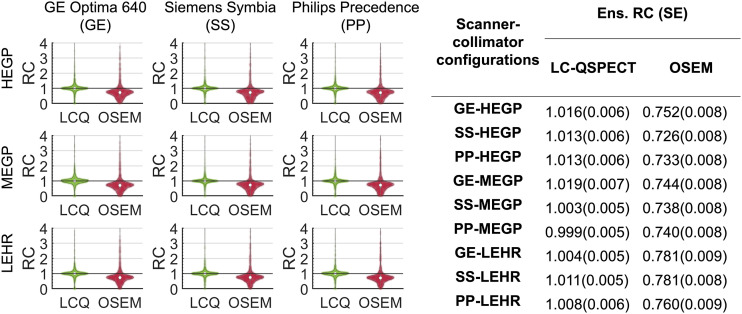
Violin plots of and ensemble-averaged RCs for estimated doses of all 2,903 lesions in trial cohort, obtained using LC-QSPECT and OSEM methods, with 9 SPECT scanner–collimator configurations. SE of each ensemble-averaged (Ens.) RC is also presented. HEGP = high-energy general-purpose; LCQ = LC-QSPECT; LEHR = low-energy high-resolution; MEGP = medium-energy general-purpose.

The distributions of the RCs of the estimated doses in all 2,903 lesions in the cohort, across the 9 SPECT scanner–collimator configurations using the LC-QSPECT and OSEM methods, are shown in Figure 5 as violin plots. Figure 5 also presents the ensemble-averaged RCs with each scanner–collimator configuration. The estimated doses from the LC-QSPECT method were nearly ensemble-unbiased, with ensemble-averaged RCs close to one for all considered scanner–collimator configurations. By comparison, the OSEM approach yielded ensemble RCs of less than one across all acquisition configurations. LC-QSPECT outperformed the OSEM method in accuracy assessment (P<0.01).

### Test–Retest Repeatability

**FIGURE 6.**
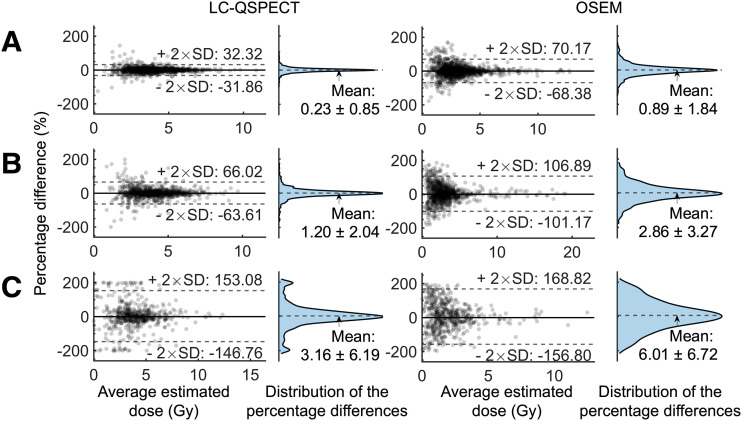
Bland–Altman plots of estimated doses for lesions with different diameter ranges: >35 mm (A), 25–35 mm (B), and <25 mm (C) in test–retest experiment. Average estimated dose was calculated by mean of first and second estimates. Vertical axis represents percentage difference, defined by difference between first and second estimates normalized by their mean. Means (±95% CI) and SDs of distributions were calculated among all data points in each group.

Figure 6 shows the Bland–Altman analyses of estimated doses for lesions in 3 diameter ranges using both LC-QSPECT and OSEM methods in the test–retest study. For both methods, the mean percentage differences showed no significant deviation from zero across all lesion diameter ranges. However, the LC-QSPECT method had a lower SD in the percentage difference between the test and retest estimates than did the OSEM method, for lesions with all diameter ranges. Furthermore, as shown in Table 3, the LC-QSPECT method yielded a significantly lower within-subject CV for all groups of VOIs (P<0.01).

**TABLE 3.** Within-Subject CV Calculated for Estimated Doses of VOIs in Each Group in Test–Retest Experiment

VOIs	LC-QSPECT	OSEM
Lesion diameter		
>35 mm	0.113	0.245
25–35 mm	0.229	0.368
<25 mm	0.530	0.577
Background	0.006	0.020
Bone	0.038	0.059
Small intestine	0.007	0.037
Large intestine	0.015	0.047

## DISCUSSION

Accurate and precise estimation of absorbed dose delivered by α-RPTs is important for their preclinical development, clinical translation, and optimal clinical application. The low number of detected photons and complex emission spectra with these therapies pose considerable challenges in using noninvasive methods to make these measures. Further, the wide variety of detector system configurations limits the comparability across data acquired from multiple centers and even within the same institution. In this ISIT-QA trial, we have studied the reproducibility across scanner–collimator configurations, accuracy, and test–retest repeatability in estimating the dose of the Food and Drug Administration–approved ^223^RaCl_2_ with LC-QSPECT and OSEM methods.

Figures 4–6 and Tables 2 and 3 demonstrate the high reproducibility across scanner–collimator configurations, accuracy, and repeatability of the LC-QSPECT method in estimating ^223^Ra-based α-RPT dose. As shown in Table 2 and Figure 4, the LC-QSPECT method yielded highly reproducible dose estimates across the ranges of acquisition configurations as demonstrated by the ICCs and outperformed the conventional OSEM method on this criterion. Across all scanner–collimator configurations, the average ensemble-averaged CVs across the 3 groups of lesions were 0.20 and 0.36 for the LC-QSPECT and OSEM methods, respectively (Fig. 4). These findings are further validated by our physical-phantom study (National Electrical Manufacturers Association phantom) detailed in Supplemental Section 7 and Supplemental Figures 4 and 5. The significance of these results is underscored by our use of a vendor-neutral OSEM reconstruction algorithm that has been demonstrated to reduce interscanner variability in estimates compared with vendor-specific algorithms (12). Comparing the reproducibility of LC-QSPECT across scanner–collimator configurations with vendor-specific OSEM methods warrants future research given their clinical relevance. The ensemble-averaged CVs for the OSEM method were slightly higher than those reported values in previous studies that were not with α-RPTs (12–14). This is expected given the higher noise in α-SPECT due to the lower counts.

Figure 5 shows that LC-QSPECT yielded close-to-zero ensemble bias dose estimates for all considered SPECT scanner–collimator configurations, with average RCs close to 1 (range, 0.999–1.019). In contrast, ensemble-averaged RCs for the OSEM method were around 0.75 (range, 0.726–0.781). A counterintuitive observation in this figure is the narrow ranges of the ensemble-averaged RCs for both LC-QSPECT and OSEM methods, since previous research (49) indicates that the use of low-energy or medium-energy collimators rather than high-energy collimators could result in lower lesion-to-background ratios. This observation, which warrants further investigation, suggests that for regional uptake estimation, the choice of collimator may not have a significant impact on ensemble accuracy. The ability of the LC-QSPECT method to accurately and precisely quantify regional uptake in ^223^Ra-based α-RPT has been shown using a single SPECT scanner–collimator configuration in our previous work (9). Results from ISIT-QA advance these abilities to a multicenter setup by demonstrating the reliability of the LC-QSPECT method in quantifying doses across multiple SPECT scanner–collimator configurations.

In the test–retest experiment (Fig. 6; Table 3), LC-QSPECT yielded a low within-subject CV of 0.28, on average across all lesions, with very low values for large lesions and soft tissues. The SD of the percentage differences between the first and second estimates calculated across all 2,903 lesions was 39.6%. These values are considerably low given the high noise levels in the SPECT projection and are lower than those from the OSEM method (within-subject CV, 0.37; SD, 52.6%).

The reliable performance of the LC-QSPECT method on the task of measuring dose, as demonstrated in ISIT-QA, is of high clinical significance. Personalized dosimetry, as observed with conventional radiopharmaceutical therapies, has been shown to enhance patient outcomes by optimizing therapeutic dose levels to balance treatment efficacy and minimize adverse events (15). However, the application of personalized dosimetry and dosimetry-guided therapy in α-RPTs has been limited, primarily because of the lack of a reliable dose quantification method (50). The LC-QSPECT method, with its ease of implementation and reliable performance, provides a promising solution for reliable regional dose quantification. In this context, we recognize that microdosimetry is important in α-RPTs (51), but LC-QSPECT (and OSEM SPECT reconstruction methods in α-RPTs) cannot directly estimate uptake distribution at submillimeter resolution. However, the reliable estimates of regional uptake given by the LC-QSPECT method can serve as input to microdosimetry approaches that use pharmacokinetic models, which can model the microscale kinetics based on macrolevel measurements (51*,*^52^). Further, the high reproducibility across scanner–collimator configurations and repeatability of the LC-QSPECT method are also important in the development and evaluation of dosimetry-guided therapy models in clinical trials. These properties of the method enable comparing and combining data from multiple centers, thus increasing the robustness of the dose–response relationship prediction (14*,*^16^).

We took great care while designing ISIT-QA to ensure clinical realism at each step of the trial pipeline, including assembling the patient cohort and simulating SPECT systems. The realism of the trial is supported by the dose quantification results. As illustrated in Figure 6, the 95% interval of the mean estimated time-integrated doses for all lesions using LC-QSPECT in the test–retest study was 1.4–7.2 Gy, reflecting absorbed doses reported in clinical studies with ^223^Ra (25).

This trial did not evaluate the impact of multiple readers, nor did it evaluate the variability and inaccuracy in the segmentation of the CT images on the LC-QSPECT approach; both of these issues may be nontrivial (14) and need to be further evaluated. Furthermore, in this trial and that of Li et al. (9), virtual patients had homogeneous uptake within the VOIs, which aligns with the assumption of the LC-QSPECT. However, real-world patients may present with a heterogeneous intraregional uptake distribution. Even though the LC-QSPECT method was observed to be relatively insensitive to certain degrees of intralesional heterogeneous uptake (9) and we can intentionally segment VOIs with relatively uniform uptake, further research could investigate the implications of potential nonuniform uptake within VOIs. Additionally, because of the limited dataset of patient-specific ^223^Ra radiopharmacokinetics, we assumed a static distribution of the isotope across acquisitions and considered only its physical decay in virtual patients. Therefore, we used a simplified, single-time-point dosimetry estimate focusing primarily on assessing the impact of QSPECT methods on dose. A promising direction for future research is to incorporate models of radiopharmacokinetics into virtual patient simulations and consider this in the dose calculation, which could further enhance the realism of patient simulations and enable more advanced development and evaluation of QSPECT dose estimation techniques. Data from the ongoing clinical trial evaluating the distributions of ^223^Ra in α-RPTs (e.g., NCT04521361) might be used for this purpose in the future.

The encouraging outcomes observed in ISIT-QA provide motivation and inputs toward designing a multicenter patient trial for clinical evaluation of LC-QPSECT. On the basis of the high reproducibility across scanner–collimator configurations, accuracy, and test–retest repeatability of the LC-QSPECT seen in the ISIT-QA trial, we anticipate that the patient trial will indicate similar performance for LC-QPSECT and thus provide stronger evidence for the use of LC-QSPECT in modeling the radiopharmacokinetics of ^223^Ra and strengthening the robustness of dose–response relationship models, thereby advancing the personalization of treatment in ^223^Ra-based α-RPTs.

## CONCLUSION

Results from ISIT-QA demonstrate that a projection-domain LC-QSPECT method yielded both high reproducibility and high accuracy across multiple scanner–collimator configurations and high test–retest repeatability on the task of estimating dose for α-RPTs. Further, LC-QSPECT outperformed the conventional OSEM method on this estimation task. These results demonstrate the potential of, and motivate the multicenter clinical assessment of, dose quantification using LC-QSPECT in α-RPTs.

## DISCLOSURE

Financial support was received from National Institute of Biomedical Imaging and Bioengineering grants R01-EB031962 and R01-EB031051 and from a Society of Nuclear Medicine and Molecular Imaging 2020 Student Research grant. No other potential conflict of interest relevant to this article was reported.

## Notes

KEY POINTS **QUESTION:** How well does the projection-domain LC-QSPECT method perform in dose quantification for ^223^Ra-based α-RPTs across multiple SPECT scanner–collimator configurations within an in silico clinical trial setting? **PERTINENT FINDINGS:** ISIT-QA showed that LC-QSPECT provided high reproducibility and accuracy across multiple scanner–collimator configurations and high test–retest repeatability on the dose estimation task, outperforming the conventional reconstruction-based quantification method. **IMPLICATIONS FOR PATIENT CARE:** The LC-QSPECT method holds promise for application in multicenter clinical trials and for helping personalize α-RPTs.
